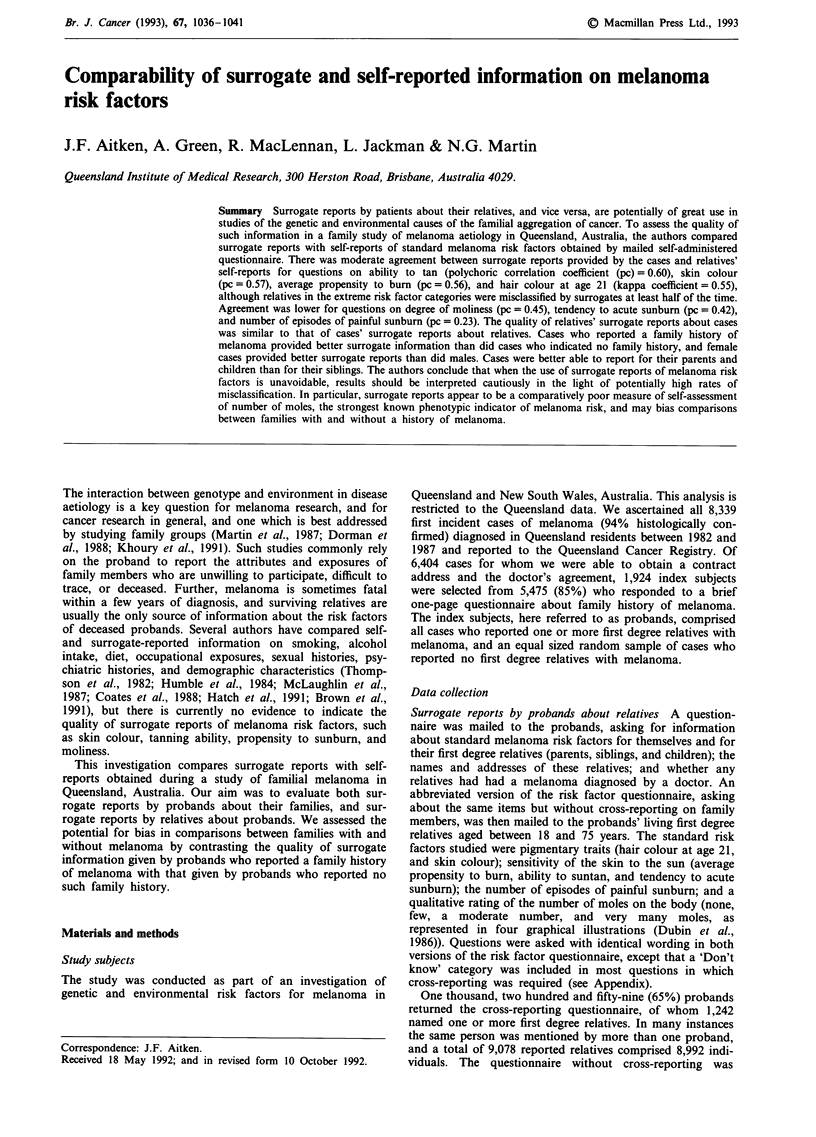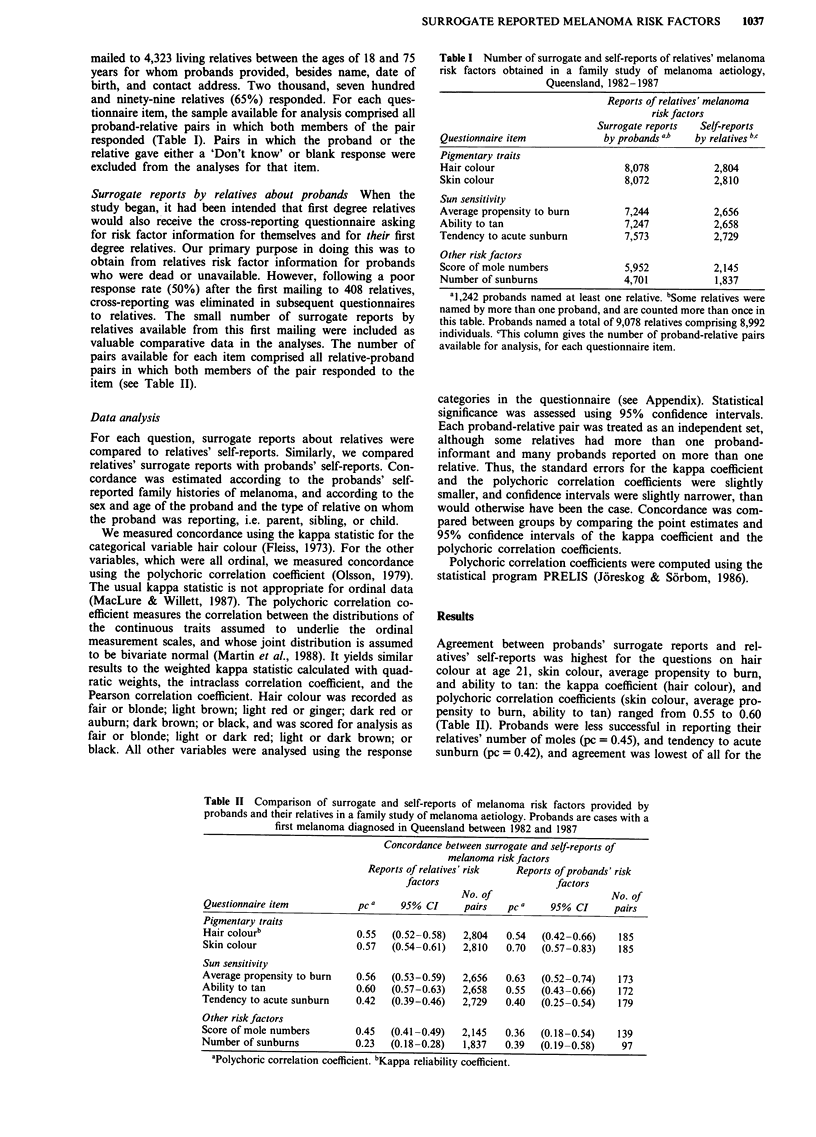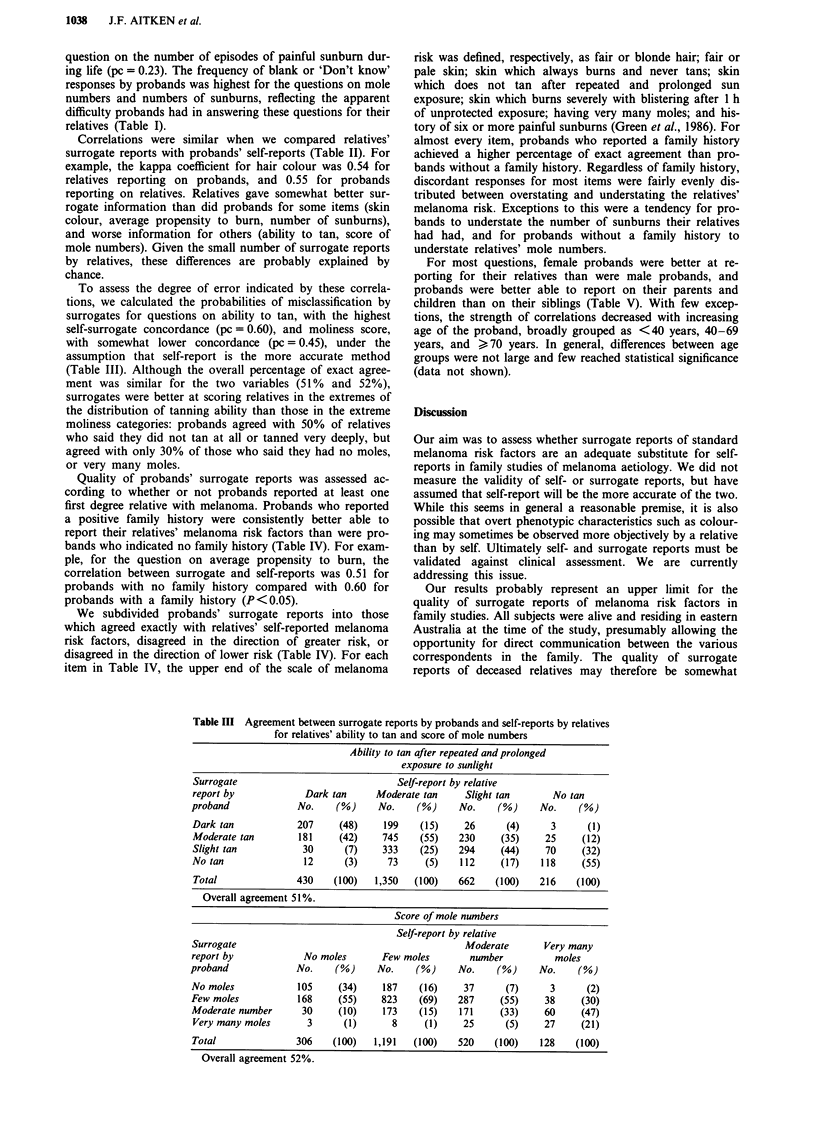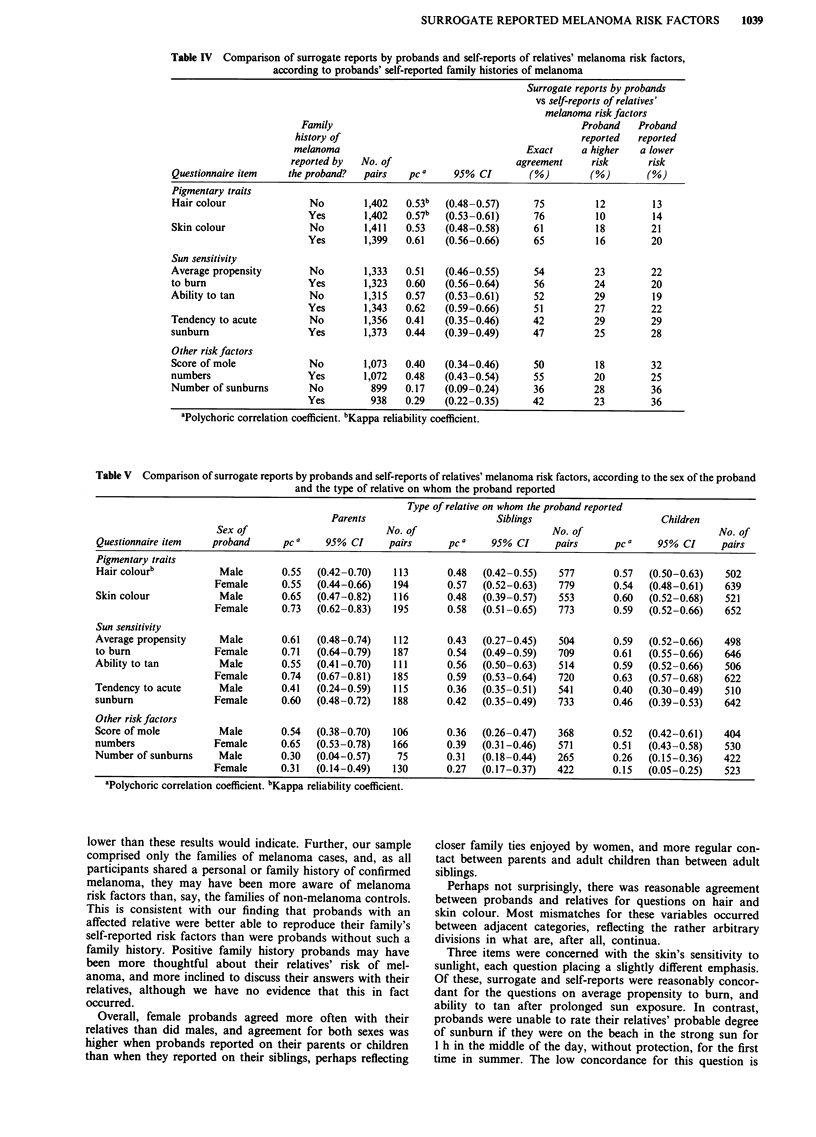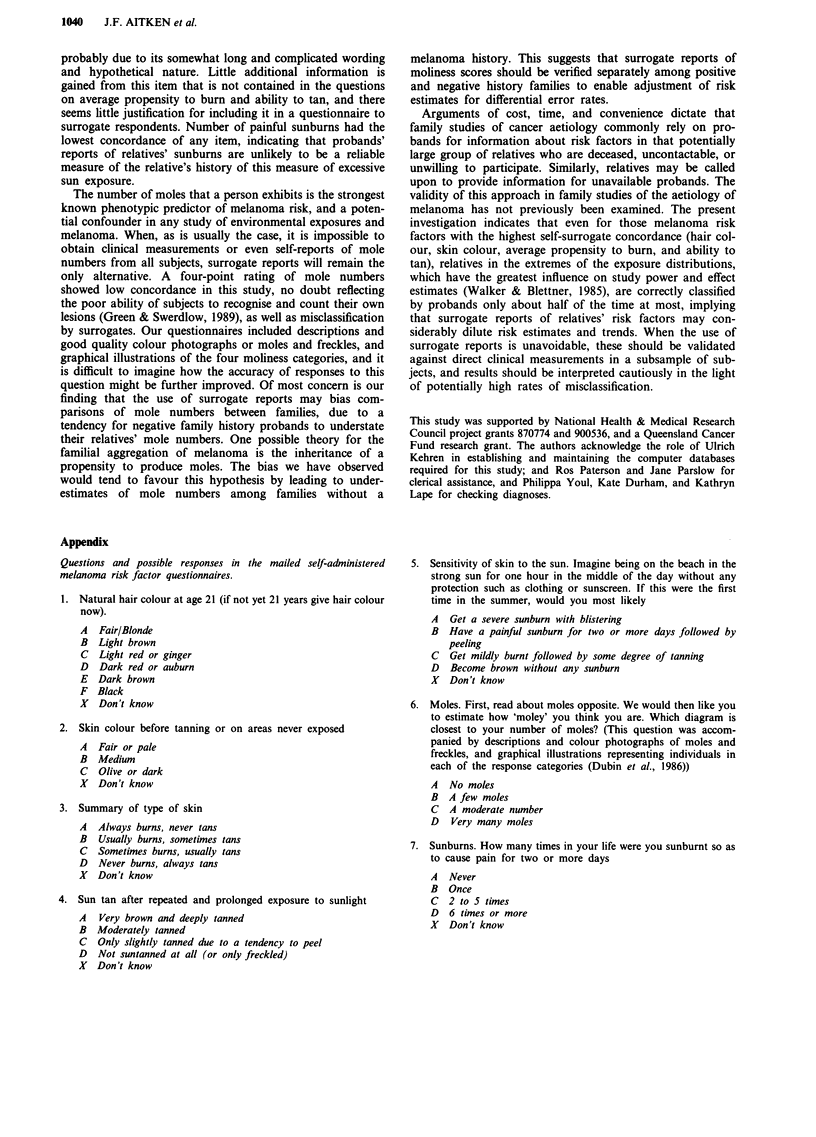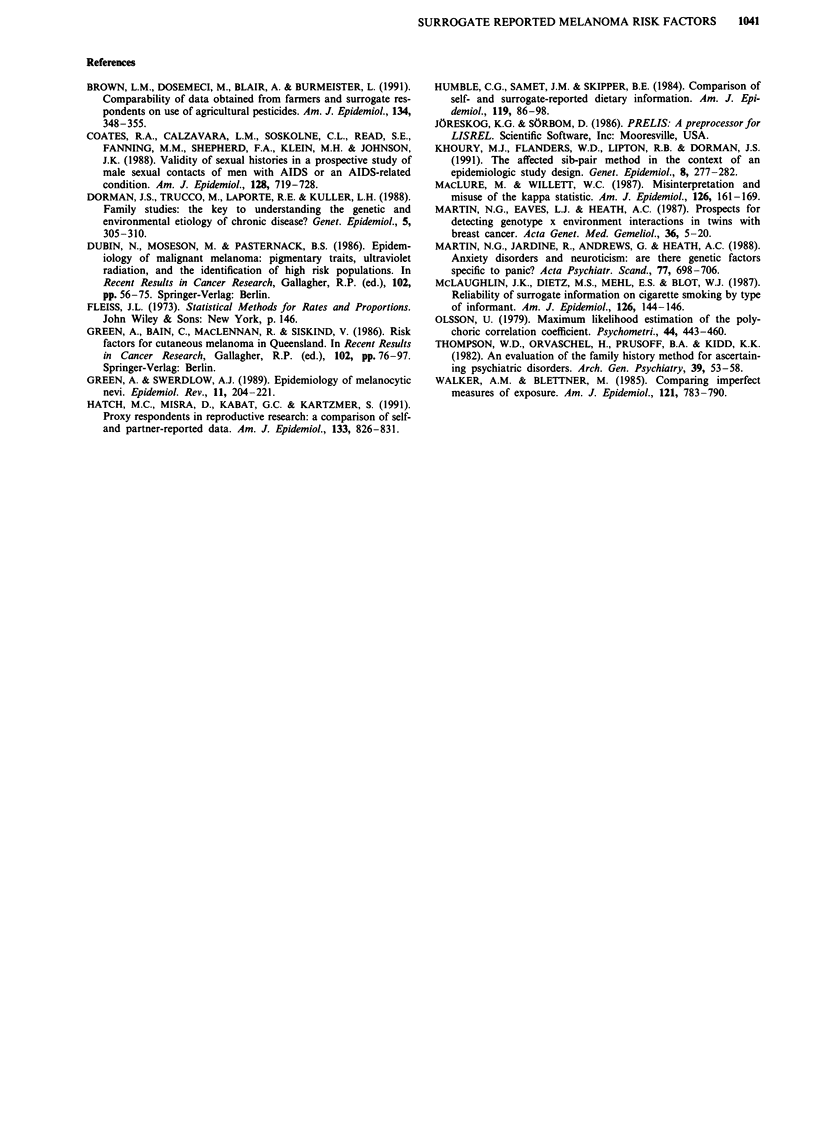# Comparability of surrogate and self-reported information on melanoma risk factors.

**DOI:** 10.1038/bjc.1993.190

**Published:** 1993-05

**Authors:** J. F. Aitken, A. Green, R. MacLennan, L. Jackman, N. G. Martin

**Affiliations:** Queensland Institute of Medical Research, Brisbane, Australia.

## Abstract

Surrogate reports by patients about their relatives, and vice versa, are potentially of great use in studies of the genetic and environmental causes of the familial aggregation of cancer. To assess the quality of such information in a family study of melanoma aetiology in Queensland, Australia, the authors compared surrogate reports with self-reports of standard melanoma risk factors obtained by mailed self-administered questionnaire. There was moderate agreement between surrogate reports provided by the cases and relatives' self-reports for questions on ability to tan (polychoric correlation coefficient (pc) = 0.60), skin colour (pc = 0.57), average propensity to burn (pc = 0.56), and hair colour at age 21 (kappa coefficient = 0.55), although relatives in the extreme risk factor categories were misclassified by surrogates at least half of the time. Agreement was lower for questions on degree of moliness (pc = 0.45), tendency to acute sunburn (pc = 0.42), and number of episodes of painful sunburn (pc = 0.23). The quality of relatives' surrogate reports about cases was similar to that of cases' surrogate reports about relatives. Cases who reported a family history of melanoma provided better surrogate information than did cases who indicated no family history, and female cases provided better surrogate reports than did males. Cases were better able to report for their parents and children than for their siblings. The authors conclude that when the use of surrogate reports of melanoma risk factors is unavoidable, results should be interpreted cautiously in the light of potentially high rates of misclassification. In particular, surrogate reports appear to be a comparatively poor measure of self-assessment of number of moles, the strongest known phenotypic indicator of melanoma risk, and may bias comparisons between families with and without a history of melanoma.


© Macmillan Press Ltd., 1993

### Comparability of surrogate and self-reported information on melanoma risk factors

J.F. Aitken, A. Green, R. MacLennan, L. Jackman & N.G. Martin

*Queensland Institute of Medical Research, 300 Herston Road, Brisbane, Australia 4029.*

**Summary** Surrogate reports by patients about their relatives, and vice versa, are potentially of great use in studies of the genetic and environmental causes of the familial aggregation of cancer. To assess the quality of such information in a family study of melanoma aetiology in Queensland, Australia, the authors compared surrogate reports with self-reports of standard melanoma risk factors obtained by mailed self-administered questionnaire. There was moderate agreement between surrogate reports provided by the cases and relatives' self-reports for questions on ability to tan (polychoric correlation coefficient (pc) = 0.60), skin colour (pc = 0.57), average propensity to burn (pc = 0.56), and hair colour at age 21 (kappa coefficient = 0.55), although relatives in the extreme risk factor categories were misclassified by surrogates at least half of the time. Agreement was lower for questions on degree of moliness (pc = 0.45), tendency to acute sunburn (pc = 0.42), and number of episodes of painful sunburn (pc = 0.23). The quality of relatives' surrogate reports about cases was similar to that of cases' surrogate reports about relatives. Cases who reported a family history of melanoma provided better surrogate information than did cases who indicated no family history, and female cases provided better surrogate reports than did males. Cases were better able to report for their parents and children than for their siblings. The authors conclude that when the use of surrogate reports of melanoma risk factors is unavoidable, results should be interpreted cautiously in the light of potentially high rates of misclassification. In particular, surrogate reports appear to be a comparatively poor measure of self-assessment of number of moles, the strongest known phenotypic indicator of melanoma risk, and may bias comparisons between families with and without a history of melanoma.

The interaction between genotype and environment in disease aetiology is a key question for melanoma research, and for cancer research in general, and one which is best addressed by studying family groups (Martin *et al.*, 1987; Dorman *et al.*, 1988; Khoury *et al.*, 1991). Such studies commonly rely on the proband to report the attributes and exposures of family members who are unwilling to participate, difficult to trace, or deceased. Further, melanoma is sometimes fatal within a few years of diagnosis, and surviving relatives are usually the only source of information about the risk factors of deceased probands. Several authors have compared self- and surrogate-reported information on smoking, alcohol intake, diet, occupational exposures, sexual histories, psychiatric histories, and demographic characteristics (Thompson *et al.*, 1982; Humble *et al.*, 1984; McLaughlin *et al.*, 1987; Coates *et al.*, 1988; Hatch *et al.*, 1991; Brown *et al.*, 1991), but there is currently no evidence to indicate the quality of surrogate reports of melanoma risk factors, such as skin colour, tanning ability, propensity to sunburn, and moliness.

This investigation compares surrogate reports with self-reports obtained during a study of familial melanoma in Queensland, Australia. Our aim was to evaluate both surrogate reports by probands about their families, and surrogate reports by relatives about probands. We assessed the potential for bias in comparisons between families with and without melanoma by contrasting the quality of surrogate information given by probands who reported a family history of melanoma with that given by probands who reported no such family history.

### Materials and methods

### *Study subjects*

The study was conducted as part of an investigation of genetic and environmental risk factors for melanoma in Queensland and New South Wales, Australia. This analysis is restricted to the Queensland data. We ascertained all 8,339 first incident cases of melanoma (94% histologically confirmed) diagnosed in Queensland residents between 1982 and 1987 and reported to the Queensland Cancer Registry. Of 6,404 cases for whom we were able to obtain a contract address and the doctor's agreement, 1,924 index subjects were selected from 5,475 (85%) who responded to a brief one-page questionnaire about family history of melanoma. The index subjects, here referred to as probands, comprised all cases who reported one or more first degree relatives with melanoma, and an equal sized random sample of cases who reported no first degree relatives with melanoma.

### *Data collection*

*Surrogate reports by probands about relatives* A questionnaire was mailed to the probands, asking for information about standard melanoma risk factors for themselves and for their first degree relatives (parents, siblings, and children); the names and addresses of these relatives; and whether any relatives had had a melanoma diagnosed by a doctor. An abbreviated version of the risk factor questionnaire, asking about the same items but without cross-reporting on family members, was then mailed to the probands' living first degree relatives aged between 18 and 75 years. The standard risk factors studied were pigmentary traits (hair colour at age 21, and skin colour); sensitivity of the skin to the sun (average propensity to burn, ability to suntan, and tendency to acute sunburn); the number of episodes of painful sunburn; and a qualitative rating of the number of moles on the body (none, few, a moderate number, and very many moles, as represented in four graphical illustrations (Dubin *et al.*, 1986)). Questions were asked with identical wording in both versions of the risk factor questionnaire, except that a 'Don't know' category was included in most questions in which cross-reporting was required (see Appendix).

One thousand, two hundred and fifty-nine (65%) probands returned the cross-reporting questionnaire, of whom 1,242 named one or more first degree relatives. In many instances the same person was mentioned by more than one proband, and a total of 9,078 reported relatives comprised 8,992 individuals. The questionnaire without cross-reporting was

Correspondence: J.F. Aitken.
Received 18 May 1992; and in revised form 10 October 1992.

mailed to 4,323 living relatives between the ages of 18 and 75 years for whom probands provided, besides name, date of birth, and contact address. Two thousand, seven hundred and ninety-nine relatives (65%) responded. For each questionnaire item, the sample available for analysis comprised all proband-relative pairs in which both members of the pair responded (Table I). Pairs in which the proband or the relative gave either a 'Don't know' or blank response were excluded from the analyses for that item.

*Surrogate reports by relatives about probands* When the study began, it had been intended that first degree relatives would also receive the cross-reporting questionnaire asking for risk factor information for themselves and for *their* first degree relatives. Our primary purpose in doing this was to obtain from relatives risk factor information for probands who were dead or unavailable. However, following a poor response rate (50%) after the first mailing to 408 relatives, cross-reporting was eliminated in subsequent questionnaires to relatives. The small number of surrogate reports by relatives available from this first mailing were included as valuable comparative data in the analyses. The number of pairs available for each item comprised all relative-proband pairs in which both members of the pair responded to the item (see Table II).

### *Data analysis*

For each question, surrogate reports about relatives were compared to relatives' self-reports. Similarly, we compared relatives' surrogate reports with probands' self-reports. Concordance was estimated according to the probands' self-reported family histories of melanoma, and according to the sex and age of the proband and the type of relative on whom the proband was reporting, i.e. parent, sibling, or child.

We measured concordance using the kappa statistic for the categorical variable hair colour (Fleiss, 1973). For the other variables, which were all ordinal, we measured concordance using the polychoric correlation coefficient (Olsson, 1979). The usual kappa statistic is not appropriate for ordinal data (MacLure & Willett, 1987). The polychoric correlation coefficient measures the correlation between the distributions of the continuous traits assumed to underlie the ordinal measurement scales, and whose joint distribution is assumed to be bivariate normal (Martin *et al.*, 1988). It yields similar results to the weighted kappa statistic calculated with quadratic weights, the intraclass correlation coefficient, and the Pearson correlation coefficient. Hair colour was recorded as fair or blonde; light brown; light red or ginger; dark red or auburn; dark brown; or black, and was scored for analysis as fair or blonde; light or dark red; light or dark brown; or black. All other variables were analysed using the response categories in the questionnaire (see Appendix). Statistical significance was assessed using 95% confidence intervals. Each proband-relative pair was treated as an independent set, although some relatives had more than one proband-informant and many probands reported on more than one relative. Thus, the standard errors for the kappa coefficient and the polychoric correlation coefficients were slightly smaller, and confidence intervals were slightly narrower, than would otherwise have been the case. Concordance was compared between groups by comparing the point estimates and 95% confidence intervals of the kappa coefficient and the polychoric correlation coefficients.

Polychoric correlation coefficients were computed using the statistical program PRELIS (Jöreskog & Sörbom, 1986).

**Table I** Number of surrogate and self-reports of relatives' melanoma risk factors obtained in a family study of melanoma aetiology, Queensland, 1982–1987

| Questionnaire item | Reports of relatives' melanoma risk factors: Surrogate reports by probands [a,b] | Self-reports by relatives [b,c] |
|---|---|---|
| *Pigmentary traits* | | |
| Hair colour | 8,078 | 2,804 |
| Skin colour | 8,072 | 2,810 |
| *Sun sensitivity* | | |
| Average propensity to burn | 7,244 | 2,656 |
| Ability to tan | 7,247 | 2,658 |
| Tendency to acute sunburn | 7,573 | 2,729 |
| *Other risk factors* | | |
| Score of mole numbers | 5,952 | 2,145 |
| Number of sunburns | 4,701 | 1,837 |

[a]1,242 probands named at least one relative. [b]Some relatives were named by more than one proband, and are counted more than once in this table. Probands named a total of 9,078 relatives comprising 8,992 individuals. [c]This column gives the number of proband-relative pairs available for analysis, for each questionnaire item.

### Results

Agreement between probands' surrogate reports and relatives' self-reports was highest for the questions on hair colour at age 21, skin colour, average propensity to burn, and ability to tan: the kappa coefficient (hair colour), and polychoric correlation coefficients (skin colour, average propensity to burn, ability to tan) ranged from 0.55 to 0.60 (Table II). Probands were less successful in reporting their relatives' number of moles (pc = 0.45), and tendency to acute sunburn (pc = 0.42), and agreement was lowest of all for the

**Table II** Comparison of surrogate and self-reports of melanoma risk factors provided by probands and their relatives in a family study of melanoma aetiology. Probands are cases with a first melanoma diagnosed in Queensland between 1982 and 1987

| Questionnaire item | Reports of relatives' risk factors: pc [a] | 95% CI | No. of pairs | Reports of probands' risk factors: pc [a] | 95% CI | No. of pairs |
|---|---|---|---|---|---|---|
| *Pigmentary traits* | | | | | | |
| Hair colour[b] | 0.55 | (0.52–0.58) | 2,804 | 0.54 | (0.42–0.66) | 185 |
| Skin colour | 0.57 | (0.54–0.61) | 2,810 | 0.70 | (0.57–0.83) | 185 |
| *Sun sensitivity* | | | | | | |
| Average propensity to burn | 0.56 | (0.53–0.59) | 2,656 | 0.63 | (0.52–0.74) | 173 |
| Ability to tan | 0.60 | (0.57–0.63) | 2,658 | 0.55 | (0.43–0.66) | 172 |
| Tendency to acute sunburn | 0.42 | (0.39–0.46) | 2,729 | 0.40 | (0.25–0.54) | 179 |
| *Other risk factors* | | | | | | |
| Score of mole numbers | 0.45 | (0.41–0.49) | 2,145 | 0.36 | (0.18–0.54) | 139 |
| Number of sunburns | 0.23 | (0.18–0.28) | 1,837 | 0.39 | (0.19–0.58) | 97 |

Concordance between surrogate and self-reports of melanoma risk factors.

[a]Polychoric correlation coefficient. [b]Kappa reliability coefficient.

question on the number of episodes of painful sunburn during life (pc = 0.23). The frequency of blank or 'Don't know' responses by probands was highest for the questions on mole numbers and numbers of sunburns, reflecting the apparent difficulty probands had in answering these questions for their relatives (Table I).

Correlations were similar when we compared relatives' surrogate reports with probands' self-reports (Table II). For example, the kappa coefficient for hair colour was 0.54 for relatives reporting on probands, and 0.55 for probands reporting on relatives. Relatives gave somewhat better surrogate information than did probands for some items (skin colour, average propensity to burn, number of sunburns), and worse information for others (ability to tan, score of mole numbers). Given the small number of surrogate reports by relatives, these differences are probably explained by chance.

To assess the degree of error indicated by these correlations, we calculated the probabilities of misclassification by surrogates for questions on ability to tan, with the highest self-surrogate concordance (pc = 0.60), and moliness score, with somewhat lower concordance (pc = 0.45), under the assumption that self-report is the more accurate method (Table III). Although the overall percentage of exact agreement was similar for the two variables (51% and 52%), surrogates were better at scoring relatives in the extremes of the distribution of tanning ability than those in the extreme moliness categories: probands agreed with 50% of relatives who said they did not tan at all or tanned very deeply, but agreed with only 30% of those who said they had no moles, or very many moles.

Quality of probands' surrogate reports was assessed according to whether or not probands reported at least one first degree relative with melanoma. Probands who reported a positive family history were consistently better able to report their relatives' melanoma risk factors than were probands who indicated no family history (Table IV). For example, for the question on average propensity to burn, the correlation between surrogate and self-reports was 0.51 for probands with no family history compared with 0.60 for probands with a family history ($P<0.05$).

We subdivided probands' surrogate reports into those which agreed exactly with relatives' self-reported melanoma risk factors, disagreed in the direction of greater risk, or disagreed in the direction of lower risk (Table IV). For each item in Table IV, the upper end of the scale of melanoma risk was defined, respectively, as fair or blonde hair; fair or pale skin; skin which always burns and never tans; skin which does not tan after repeated and prolonged sun exposure; skin which burns severely with blistering after 1 h of unprotected exposure; having very many moles; and history of six or more painful sunburns (Green et al., 1986). For almost every item, probands who reported a family history achieved a higher percentage of exact agreement than probands without a family history. Regardless of family history, discordant responses for most items were fairly evenly distributed between overstating and understating the relatives' melanoma risk. Exceptions to this were a tendency for probands to understate the number of sunburns their relatives had had, and for probands without a family history to understate relatives' mole numbers.

For most questions, female probands were better at reporting for their relatives than were male probands, and probands were better able to report on their parents and children than on their siblings (Table V). With few exceptions, the strength of correlations decreased with increasing age of the proband, broadly grouped as <40 years, 40–69 years, and ≥70 years. In general, differences between age groups were not large and few reached statistical significance (data not shown).

### Discussion

Our aim was to assess whether surrogate reports of standard melanoma risk factors are an adequate substitute for self-reports in family studies of melanoma aetiology. We did not measure the validity of self- or surrogate reports, but have assumed that self-report will be the more accurate of the two. While this seems in general a reasonable premise, it is also possible that overt phenotypic characteristics such as colouring may sometimes be observed more objectively by a relative than by self. Ultimately self- and surrogate reports must be validated against clinical assessment. We are currently addressing this issue.

Our results probably represent an upper limit for the quality of surrogate reports of melanoma risk factors in family studies. All subjects were alive and residing in eastern Australia at the time of the study, presumably allowing the opportunity for direct communication between the various correspondents in the family. The quality of surrogate reports of deceased relatives may therefore be somewhat

**Table III** Agreement between surrogate reports by probands and self-reports by relatives for relatives' ability to tan and score of mole numbers

*Ability to tan after repeated and prolonged exposure to sunlight*

| *Surrogate report by proband* | *Dark tan* No. | (%) | *Moderate tan* No. | (%) | *Slight tan* No. | (%) | *No tan* No. | (%) |
|---|---|---|---|---|---|---|---|---|
| | *Self-report by relative* | | | | | | | |
| *Dark tan* | 207 | (48) | 199 | (15) | 26 | (4) | 3 | (1) |
| *Moderate tan* | 181 | (42) | 745 | (55) | 230 | (35) | 25 | (12) |
| *Slight tan* | 30 | (7) | 333 | (25) | 294 | (44) | 70 | (32) |
| *No tan* | 12 | (3) | 73 | (5) | 112 | (17) | 118 | (55) |
| *Total* | 430 | (100) | 1,350 | (100) | 662 | (100) | 216 | (100) |

Overall agreement 51%.

*Score of mole numbers*

| *Surrogate report by proband* | *No moles* No. | (%) | *Few moles* No. | (%) | *Moderate number* No. | (%) | *Very many moles* No. | (%) |
|---|---|---|---|---|---|---|---|---|
| | *Self-report by relative* | | | | | | | |
| *No moles* | 105 | (34) | 187 | (16) | 37 | (7) | 3 | (2) |
| *Few moles* | 168 | (55) | 823 | (69) | 287 | (55) | 38 | (30) |
| *Moderate number* | 30 | (10) | 173 | (15) | 171 | (33) | 60 | (47) |
| *Very many moles* | 3 | (1) | 8 | (1) | 25 | (5) | 27 | (21) |
| *Total* | 306 | (100) | 1,191 | (100) | 520 | (100) | 128 | (100) |

Overall agreement 52%.

**Table IV** Comparison of surrogate reports by probands and self-reports of relatives' melanoma risk factors, according to probands' self-reported family histories of melanoma

| | | | | | *Surrogate reports by probands vs self-reports of relatives' melanoma risk factors* | | |
|---|---|---|---|---|---|---|---|
| *Questionnaire item* | *Family history of melanoma reported by the proband?* | *No. of pairs* | *pc*[a] | *95% CI* | *Exact agreement (%)* | *Proband reported a higher risk (%)* | *Proband reported a lower risk (%)* |
| *Pigmentary traits* | | | | | | | |
| Hair colour | No | 1,402 | 0.53[b] | (0.48–0.57) | 75 | 12 | 13 |
| | Yes | 1,402 | 0.57[b] | (0.53–0.61) | 76 | 10 | 14 |
| Skin colour | No | 1,411 | 0.53 | (0.48–0.58) | 61 | 18 | 21 |
| | Yes | 1,399 | 0.61 | (0.56–0.66) | 65 | 16 | 20 |
| *Sun sensitivity* | | | | | | | |
| Average propensity | No | 1,333 | 0.51 | (0.46–0.55) | 54 | 23 | 22 |
| to burn | Yes | 1,323 | 0.60 | (0.56–0.64) | 56 | 24 | 20 |
| Ability to tan | No | 1,315 | 0.57 | (0.53–0.61) | 52 | 29 | 19 |
| | Yes | 1,343 | 0.62 | (0.59–0.66) | 51 | 27 | 22 |
| Tendency to acute | No | 1,356 | 0.41 | (0.35–0.46) | 42 | 29 | 29 |
| sunburn | Yes | 1,373 | 0.44 | (0.39–0.49) | 47 | 25 | 28 |
| *Other risk factors* | | | | | | | |
| Score of mole | No | 1,073 | 0.40 | (0.34–0.46) | 50 | 18 | 32 |
| numbers | Yes | 1,072 | 0.48 | (0.43–0.54) | 55 | 20 | 25 |
| Number of sunburns | No | 899 | 0.17 | (0.09–0.24) | 36 | 28 | 36 |
| | Yes | 938 | 0.29 | (0.22–0.35) | 42 | 23 | 36 |

[a]Polychoric correlation coefficient. [b]Kappa reliability coefficient.

**Table V** Comparison of surrogate reports by probands and self-reports of relatives' melanoma risk factors, according to the sex of the proband and the type of relative on whom the proband reported

| | | *Type of relative on whom the proband reported* | | | | | | | | |
|---|---|---|---|---|---|---|---|---|---|---|
| | | *Parents* | | | *Siblings* | | | *Children* | | |
| *Questionnaire item* | *Sex of proband* | *pc*[a] | *95% CI* | *No. of pairs* | *pc*[a] | *95% CI* | *No. of pairs* | *pc*[a] | *95% CI* | *No. of pairs* |
| *Pigmentary traits* | | | | | | | | | | |
| Hair colour[b] | Male | 0.55 | (0.42–0.70) | 113 | 0.48 | (0.42–0.55) | 577 | 0.57 | (0.50–0.63) | 502 |
| | Female | 0.55 | (0.44–0.66) | 194 | 0.57 | (0.52–0.63) | 779 | 0.54 | (0.48–0.61) | 639 |
| Skin colour | Male | 0.65 | (0.47–0.82) | 116 | 0.48 | (0.39–0.57) | 553 | 0.60 | (0.52–0.68) | 521 |
| | Female | 0.73 | (0.62–0.83) | 195 | 0.58 | (0.51–0.65) | 773 | 0.59 | (0.52–0.66) | 652 |
| *Sun sensitivity* | | | | | | | | | | |
| Average propensity | Male | 0.61 | (0.48–0.74) | 112 | 0.43 | (0.27–0.45) | 504 | 0.59 | (0.52–0.66) | 498 |
| to burn | Female | 0.71 | (0.64–0.79) | 187 | 0.54 | (0.49–0.59) | 709 | 0.61 | (0.55–0.66) | 646 |
| Ability to tan | Male | 0.55 | (0.41–0.70) | 111 | 0.56 | (0.50–0.63) | 514 | 0.59 | (0.52–0.66) | 506 |
| | Female | 0.74 | (0.67–0.81) | 185 | 0.59 | (0.53–0.64) | 720 | 0.63 | (0.57–0.68) | 622 |
| Tendency to acute | Male | 0.41 | (0.24–0.59) | 115 | 0.36 | (0.35–0.51) | 541 | 0.40 | (0.30–0.49) | 510 |
| sunburn | Female | 0.60 | (0.48–0.72) | 188 | 0.42 | (0.35–0.49) | 733 | 0.46 | (0.39–0.53) | 642 |
| *Other risk factors* | | | | | | | | | | |
| Score of mole | Male | 0.54 | (0.38–0.70) | 106 | 0.36 | (0.26–0.47) | 368 | 0.52 | (0.42–0.61) | 404 |
| numbers | Female | 0.65 | (0.53–0.78) | 166 | 0.39 | (0.31–0.46) | 571 | 0.51 | (0.43–0.58) | 530 |
| Number of sunburns | Male | 0.30 | (0.04–0.57) | 75 | 0.31 | (0.18–0.44) | 265 | 0.26 | (0.15–0.36) | 422 |
| | Female | 0.31 | (0.14–0.49) | 130 | 0.27 | (0.17–0.37) | 422 | 0.15 | (0.05–0.25) | 523 |

[a]Polychoric correlation coefficient. [b]Kappa reliability coefficient.

lower than these results would indicate. Further, our sample comprised only the families of melanoma cases, and, as all participants shared a personal or family history of confirmed melanoma, they may have been more aware of melanoma risk factors than, say, the families of non-melanoma controls. This is consistent with our finding that probands with an affected relative were better able to reproduce their family's self-reported risk factors than were probands without such a family history. Positive family history probands may have been more thoughtful about their relatives' risk of melanoma, and more inclined to discuss their answers with their relatives, although we have no evidence that this in fact occurred.

Overall, female probands agreed more often with their relatives than did males, and agreement for both sexes was higher when probands reported on their parents or children than when they reported on their siblings, perhaps reflecting closer family ties enjoyed by women, and more regular contact between parents and adult children than between adult siblings.

Perhaps not surprisingly, there was reasonable agreement between probands and relatives for questions on hair and skin colour. Most mismatches for these variables occurred between adjacent categories, reflecting the rather arbitrary divisions in what are, after all, continua.

Three items were concerned with the skin's sensitivity to sunlight, each question placing a slightly different emphasis. Of these, surrogate and self-reports were reasonably concordant for the questions on average propensity to burn, and ability to tan after prolonged sun exposure. In contrast, probands were unable to rate their relatives' probable degree of sunburn if they were on the beach in the strong sun for 1 h in the middle of the day, without protection, for the first time in summer. The low concordance for this question is probably due to its somewhat long and complicated wording and hypothetical nature. Little additional information is gained from this item that is not contained in the questions on average propensity to burn and ability to tan, and there seems little justification for including it in a questionnaire to surrogate respondents. Number of painful sunburns had the lowest concordance of any item, indicating that probands' reports of relatives' sunburns are unlikely to be a reliable measure of the relative's history of this measure of excessive sun exposure.

The number of moles that a person exhibits is the strongest known phenotypic predictor of melanoma risk, and a potential confounder in any study of environmental exposures and melanoma. When, as is usually the case, it is impossible to obtain clinical measurements or even self-reports of mole numbers from all subjects, surrogate reports will remain the only alternative. A four-point rating of mole numbers showed low concordance in this study, no doubt reflecting the poor ability of subjects to recognise and count their own lesions (Green & Swerdlow, 1989), as well as misclassification by surrogates. Our questionnaires included descriptions and good quality colour photographs or moles and freckles, and graphical illustrations of the four moliness categories, and it is difficult to imagine how the accuracy of responses to this question might be further improved. Of most concern is our finding that the use of surrogate reports may bias comparisons of mole numbers between families, due to a tendency for negative family history probands to understate their relatives' mole numbers. One possible theory for the familial aggregation of melanoma is the inheritance of a propensity to produce moles. The bias we have observed would tend to favour this hypothesis by leading to underestimates of mole numbers among families without a melanoma history. This suggests that surrogate reports of moliness scores should be verified separately among positive and negative history families to enable adjustment of risk estimates for differential error rates.

Arguments of cost, time, and convenience dictate that family studies of cancer aetiology commonly rely on probands for information about risk factors in that potentially large group of relatives who are deceased, uncontactable, or unwilling to participate. Similarly, relatives may be called upon to provide information for unavailable probands. The validity of this approach in family studies of the aetiology of melanoma has not previously been examined. The present investigation indicates that even for those melanoma risk factors with the highest self-surrogate concordance (hair colour, skin colour, average propensity to burn, and ability to tan), relatives in the extremes of the exposure distributions, which have the greatest influence on study power and effect estimates (Walker & Blettner, 1985), are correctly classified by probands only about half of the time at most, implying that surrogate reports of relatives' risk factors may considerably dilute risk estimates and trends. When the use of surrogate reports is unavoidable, these should be validated against direct clinical measurements in a subsample of subjects, and results should be interpreted cautiously in the light of potentially high rates of misclassification.

This study was supported by National Health & Medical Research Council project grants 870774 and 900536, and a Queensland Cancer Fund research grant. The authors acknowledge the role of Ulrich Kehren in establishing and maintaining the computer databases required for this study; and Ros Paterson and Jane Parslow for clerical assistance, and Philippa Youl, Kate Durham, and Kathryn Lape for checking diagnoses.

### Appendix

*Questions and possible responses in the mailed self-administered melanoma risk factor questionnaires.*

1. Natural hair colour at age 21 (if not yet 21 years give hair colour now).

   *A Fair/Blonde*

   *B Light brown*

   *C Light red or ginger*

   *D Dark red or auburn*

   *E Dark brown*

   *F Black*

   *X Don't know*

2. Skin colour before tanning or on areas never exposed

   *A Fair or pale*

   *B Medium*

   *C Olive or dark*

   *X Don't know*

3. Summary of type of skin

   *A Always burns, never tans*

   *B Usually burns, sometimes tans*

   *C Sometimes burns, usually tans*

   *D Never burns, always tans*

   *X Don't know*

4. Sun tan after repeated and prolonged exposure to sunlight

   *A Very brown and deeply tanned*

   *B Moderately tanned*

   *C Only slightly tanned due to a tendency to peel*

   *D Not suntanned at all (or only freckled)*

   *X Don't know*

5. Sensitivity of skin to the sun. Imagine being on the beach in the strong sun for one hour in the middle of the day without any protection such as clothing or sunscreen. If this were the first time in the summer, would you most likely

   *A Get a severe sunburn with blistering*
   *B Have a painful sunburn for two or more days followed by peeling*
   *C Get mildly burnt followed by some degree of tanning*
   *D Become brown without any sunburn*
   *X Don't know*

6. Moles. First, read about moles opposite. We would then like you to estimate how 'moley' you think you are. Which diagram is closest to your number of moles? (This question was accompanied by descriptions and colour photographs of moles and freckles, and graphical illustrations representing individuals in each of the response categories (Dubin *et al.*, 1986))

   *A No moles*
   *B A few moles*
   *C A moderate number*
   *D Very many moles*

7. Sunburns. How many times in your life were you sunburnt so as to cause pain for two or more days

   *A Never*
   *B Once*
   *C 2 to 5 times*
   *D 6 times or more*
   *X Don't know*

### References

BROWN, L.M., DOSEMECI, M., BLAIR, A. & BURMEISTER, L. (1991). Comparability of data obtained from farmers and surrogate respondents on use of agricultural pesticides. *Am. J. Epidemiol.*, **134**, 348–355.

COATES, R.A., CALZAVARA, L.M., SOSKOLNE, C.L., READ, S.E., FANNING, M.M., SHEPHERD, F.A., KLEIN, M.H. & JOHNSON, J.K. (1988). Validity of sexual histories in a prospective study of male sexual contacts of men with AIDS or an AIDS-related condition. *Am. J. Epidemiol.*, **128**, 719–728.

DORMAN, J.S., TRUCCO, M., LAPORTE, R.E. & KULLER, L.H. (1988). Family studies: the key to understanding the genetic and environmental etiology of chronic disease? *Genet. Epidemiol.*, **5**, 305–310.

DUBIN, N., MOSESON, M. & PASTERNACK, B.S. (1986). Epidemiology of malignant melanoma: pigmentary traits, ultraviolet radiation, and the identification of high risk populations. In *Recent Results in Cancer Research*, Gallagher, R.P. (ed.), **102, pp.** 56–75. Springer-Verlag: Berlin.

FLEISS, J.L. (1973). *Statistical Methods for Rates and Proportions*. John Wiley & Sons: New York, p. 146.

GREEN, A., BAIN, C., MACLENNAN, R. & SISKIND, V. (1986). Risk factors for cutaneous melanoma in Queensland. In *Recent Results in Cancer Research*, Gallagher, R.P. (ed.), **102, pp.** 76–97. Springer-Verlag: Berlin.

GREEN, A. & SWERDLOW, A.J. (1989). Epidemiology of melanocytic nevi. *Epidemiol. Rev.*, **11**, 204–221.

HATCH, M.C., MISRA, D., KABAT, G.C. & KARTZMER, S. (1991). Proxy respondents in reproductive research: a comparison of self- and partner-reported data. *Am. J. Epidemiol.*, **133**, 826-831.

HUMBLE, C.G., SAMET, J.M. & SKIPPER, B.E. (1984). Comparison of self- and surrogate-reported dietary information. *Am. J. Epidemiol.*, **119**, 86–98.

JÖRESKOG, K.G. & SÖRBOM, D. (1986). *PRELIS: A preprocessor for LISREL*. Scientific Software, Inc: Mooresville, USA.

KHOURY, M.J., FLANDERS, W.D., LIPTON, R.B. & DORMAN, J.S. (1991). The affected sib-pair method in the context of an epidemiologic study design. *Genet. Epidemiol.*, **8**, 277–282.

MACLURE, M. & WILLETT, W.C. (1987). Misinterpretation and misuse of the kappa statistic. *Am. J. Epidemiol.*, **126**, 161–169.

MARTIN, N.G., EAVES, L.J. & HEATH, A.C. (1987). Prospects for detecting genotype x environment interactions in twins with breast cancer. *Acta Genet. Med. Gemeliol.*, **36**, 5–20.

MARTIN, N.G., JARDINE, R., ANDREWS, G. & HEATH, A.C. (1988). Anxiety disorders and neuroticism: are there genetic factors specific to panic? *Acta Psychiatr. Scand.*, **77**, 698–706.

MCLAUGHLIN, J.K., DIETZ, M.S., MEHL, E.S. & BLOT, W.J. (1987). Reliability of surrogate information on cigarette smoking by type of informant. *Am. J. Epidemiol.*, **126**, 144–146.

OLSSON, U. (1979). Maximum likelihood estimation of the polychoric correlation coefficient. *Psychometri.*, **44**, 443–460.

THOMPSON, W.D., ORVASCHEL, H., PRUSOFF, B.A. & KIDD, K.K. (1982). An evaluation of the family history method for ascertaining psychiatric disorders. *Arch. Gen. Psychiatry*, **39**, 53–58.

WALKER, A.M. & BLETTNER, M. (1985). Comparing imperfect measures of exposure. *Am. J. Epidemiol.*, **121**, 783–790.